# Tumor necrosis factor-alpha induces VCAM-1-mediated inflammation via c-Src-dependent transactivation of EGF receptors in human cardiac fibroblasts

**DOI:** 10.1186/s12929-015-0165-8

**Published:** 2015-07-15

**Authors:** Chih-Chung Lin, Chih-Shuo Pan, Chen-Yu Wang, Shiau-Wen Liu, Li-Der Hsiao, Chuen-Mao Yang

**Affiliations:** Department of Anesthetics, Chang Gung Memorial Hospital at Linkuo, Kwei-Shan, Tao-Yuan Taiwan; College of Medicine, Chang Gung University, Kwei-Shan, Tao-Yuan Taiwan; Department of Physiology, College of Medicine, Chang Gung University, Kwei-Shan, Tao-Yuan Taiwan; Department of Pharmacology and Health Aging Research Center, College of Medicine, Chang Gung University, 259 Wen-Hwa 1st Road, Kwei-Shan, Tao-Yuan Taiwan

**Keywords:** TNF-α, Cardiac fibroblasts, EGFR transactivation, VCAM-1, Monocytes adhesion

## Abstract

**Background:**

Tumor necrosis factor-α (TNF-α) is a proinflammatory cytokine and elevated in the regions of tissue injury and inflammatory diseases. The deleterious effects of TNF-α on fibroblasts may aggravate heart inflammation mediated through the up-regulation of adhesion molecules such as vascular cell adhesion molecule-1 (VCAM-1). However, the mechanisms underlying TNF-α-induced VCAM-1 expression in cardiac fibroblasts remain unknown. This study aimed to investigate the roles of TNF-α in VCAM-1 expression and its effects on human cardiac fibroblasts (HCFs).

**Results:**

The primary culture HCFs were used in this study. The results obtained with Western blotting, real time-quantitative PCR, and promoter activity analyses showed that TNF-α-induced VCAM-1 expression was mediated through TNF receptor (TNFR) 1-dependent gene up-regulation. Activation of TNFR1 by TNF-α transactivated c-Src-dependent EGF receptor (EGFR) linking to PI3K/Akt cascade, and then led to transcriptional activity of NF-κB. Moreover, the results of promoter reporter assay demonstrated that the phosphorylated p65 NF-κB turned on VCAM-1 gene expression. Subsequently, up-regulation of VCAM-1 promoted monocytes adhesion to HCFs challenged with TNF-α determined by cell adhesion assay.

**Conclusions:**

Taken together, these results indicate that in HCFs, activation of NF-κB by c-Src-mediated transactivation of EGFR/PI3K/Akt cascade is required for TNF-α-induced VCAM-1 expression. Finally, increased VCAM-1 enhances monocytes adhering to HCFs challenged with TNF-α. Understanding the mechanisms of VCAM-1 up-regulated by TNF-α on HCFs may provide rationally therapeutic interventions for heart injury or inflammatory diseases.

## Background

Heart failure, the first cause of death in the world, is generally defined as the inability of heart supplying sufficient blood volume and is a progressive and complex pathological condition [1]. The most common initiation leading to heart failure is coronary artery disease with myocardial infarction and hypertension [2, 3]. The previous study indicates that pathological fibrosis has emerged as a key target for pharmacological intervention in heart failure [4]. Several pro-inflammatory cytokines such as tumor necrosis factor-α (TNF-α) are elevated in acute myocardial injury and infarction. Chronically elevated these cytokines also have been shown to cause phenotypic and functional changes in the constituent cell types of the heart [5–8], including cell proliferation, production of the collagen extracellular matrix (ECM), generation of mediator substances by the cardiac fibroblast [9–11], and associate with heart failure [12].

TNF-α is a major cytokine in the pathogenesis of cardiac injury, promoting inflammation, apoptosis, and production of ECM [13, 14] and exerts as a potent stimulus in inflammatory responses through up-regulation of many genes, including cytokines, chemokines, proteases, cyclooxygenase, and adhesion molecules [15, 16]. Furthermore, inhibition of TNF-α-mediated pathways can reduce myocardial ischemia-reperfusion injury [13]. These results suggest that TNF-α may act as a key mediator to elicit various heart diseases. TNF-α-activated NF-κB is critical for the inflammatory processes [17, 18]. In addition, TNF-α induces the expression of inflammatory genes by activation of TNF receptor (TNFR)-mediated diverse signaling molecules, including c-Src family [19, 20]. As already shown in human cardiac fibroblasts MMP-9 expression is induced by TNF-α [16]. Furthermore, our previous data showed that TNF-α can induce MMP-9 expression through a c-Src/EFGR [6]. The c-Src family, a non-receptor tyrosine kinase, has been shown to exert as a target of G protein-coupled receptors (GPCRs) in transactivation of growth factor receptors such as epidermal growth factor receptor (EGFR) [21] and also involves in cytokine-stimulated transactivation of growth factor receptors like EGFR in several cell types [22].

In the serum of patients with non-ischemic heart failure, the levels of intercellular adhesion molecule (ICAM)-1 and vascular cell adhesion molecule (VCAM)-1 are often elevated. And the cellular infiltration is due to up-regulation of adhesion molecules including ICAM-1 and VCAM-1 to control cell adhesion and migration [23–26]. These raised levels of adhesion molecules correlate with inflammatory infiltrates in the myocardial tissue [27]. VCAM-1, which exhibits low to negligible expression in unstimulated endothelial cells, can be profoundly up-regulated after cytokine challenge. The induction of cell adhesion molecules mediates the tight adhesiveness of polymorphonuclear cells (PMNs) and thus facilitates PMNs migration across the vascular endothelial barrier [28, 29]. Up-regulation of VCAM-1 and TNF-α has been shown in the cardiac vascular endothelium from the patients with chronic heart failure [27, 30]. TNF-α-activated c-Src kinase and NF-κB can regulate VCAM-1 expression [31–33] and the soluble ICAM-1 release from osteoblast-like MC3T3-E1 cells [34]. VCAM-1 promoter also contains NF-κB binding sites which are regulated by TNF-α through c-Src-dependent pathways [19]. Therefore, cytokine-triggered VCAM-1 up-regulation in heart diseases may be the key response for the targeted leukocyte transmigration into extravascular space of inflammation [29, 35]. However, the mechanisms and effects of TNF-α on VCAM-1 expression via the activation of c-Src and NF-κB in primary human cardiac fibroblasts (HCFs) remain unclear.

In this study, we found that TNF-α induces c-Src-mediated signal activation in HCFs via TNFR1, in turn initiates the activation of EGFR, PI3K/Akt, and NF-κB. Activated NF-κB could accelerate VCAM-1 promoter activity to induce VCAM-1 expression in HCFs. Moreover, up-regulation of VCAM-1 promoted adhesion of monocytes to HCFs challenged with TNF-α. These results provide new insights into the TNF-α action, to up-regulate the VCAM-1 expression and then amplify the inflammatory responses in HCFs.

## Methods

### Materials

DMEM/F-12 medium, fetal bovine serum (FBS), TRIZOL, Lipofectamine-PLUS reagent and 2′,7′-bis-(2-carboxyethyl)-5-(and-6)-carboxyfluorescein, acetoxymethyl ester (BCECF/AM, B-1170) were from Invitrogen (Carlsbad, CA). The siRNAs for TNFR1 (NM_001065, SASI_Hs01_00033456), c-Src (NM_005417, SASI_Hs01_00097530), EGFR (NM_005228, SASI_Hs01_00215449), p110 (NM_006218, SASI_Hs01_00219339), Akt (NM_001014431, SASI_Hs01_00105954), and p65 (a subunit of NF-κB, NM_021975, SASI_Hs01_001220265) were from Sigma (St. Louis, MO). Hybond C membrane, enhanced chemiluminescence (ECL) and Western blotting detection system were from GE Healthcare Biosciences (Buckinghamshire, UK). PhosphoPlus EGFR (Thr^1068^) (#2236), Akt (Ser^473^) (#9271), and p65 NF-κB (Ser^536^) (#3031) antibody kits were from Cell Signaling (Danvers, MA). Human polyclonal antibody VCAM-1 (sc-8304), TNFR1 (sc-52739), phospho-c-Src (Tyr^139^) (sc-12928-R), c-Src (sc-18), p110 (PI3K subunit) (sc-7189), Akt (sc-8312), and p65 (sc-7151) antibodies were from Santa Cruz (Santa Cruz, CA). Vimentin (MS-129-P) was from Thermo Fisher Scientific (Fremont, CA). GAPDH was from Biogenesis (Bournemouth, UK). Peroxidase AffiniPure goat anti-rabbit IgG (111-035-003) and anti-mouse IgG (115-035-003) were from Jackson ImmunoResearch (West Grove, PA). PP1, AG1478, LY294002, SH-5, and Bay11-7082 were from Biomol (Plymouth Meeting, PA). TNF-α was from R&D Systems (Minneapolis, MN). Bicinchoninic acid (BCA) protein assay kit was from Pierce (Rockford, IL). Actinomycin D (Act. D), cycloheximide (CHI), enzymes, and other chemicals were from Sigma (St. Louis, MO).

### Human cardiac fibroblasts (HCFs) culture and treatment

Human cardiac fibroblasts (HCFs) were isolated from human heart obtained from ScienCell Research Lab (San Diego, CA, Cat. No. 6300) and then characterized by immunofluorescent staining with an antibody specific to fibronectin [36]. These cells were grown in DMEM/F-12 containing 10 % FBS, 2 mM glutamine and antibiotics (100 U/ml penicillin G, 100 μg/ml streptomycin, and 250 ng/ml fungizone) at 37 °C in a humidified 5 % CO_2_ atmosphere. When the cultures reached confluence, cells were treated with 0.05 % trypsin/0.53 mM EDTA for 1 min at 37 °C. The cell suspension was diluted with DMEM/F-12 containing 10 % FBS and 2 mM glutamine to a concentration of 2 × 10^5^ cells/ml. The cells were plated onto 12-well culture plates and made quiescent at confluence by incubation in serum-free DMEM/F-12 for 24 h, for growth arrest as previously described [9, 37], and then incubated with TNF-α (0.15, 1.5, 15, or 30 ng/ml) at 37 °C for the indicated time intervals. The XTT {sodium 3′-[1-(phenylamino-carbonyl)-3,4-tetrazolium]-bis(4-methoxy-6-nitro) benzene sulphonic acid hydrate} assay was performed to analyze the cell viability as previously described [38]. When the inhibitors were used, cells were pretreated with the inhibitor for 1 h before exposure to TNF-α. Treatment of HCFs with pharmacological inhibitors or TNF-α alone had no significant effect on cell viability determined by XTT assay (data not shown). Experiments were performed using cells from passages 3 to 8.

### Preparation of cell extracts and Western blot analysis

Growth-arrested HCFs (incubation in serum-free DMEM/F-12 for 24 h) were incubated with TNF-α at 37 °C for the indicated time intervals. The cells were washed with ice-cold PBS, scraped, and collected by centrifugation at 45,000 × g for 1 h at 4 °C to yield the whole cell extract, as previously described [33]. Samples were denatured, subjected to SDS-PAGE using a 10 % (w/v) running gel, and transferred to nitrocellulose membrane. Membranes were incubated overnight using a phospho-c-Src, phospho-EGFR, phospho-Akt, phospho-p65, TNFR1, EGFR, p110, Akt, p65, or GAPDH antibody. Membranes were washed with TTBS four times for 5 min each, incubated with a 1:2000 dilution of anti-rabbit horseradish peroxidase antibody for 1 h. The immunoreactive bands were detected by ECL reagents and captured by a UVP BioSpectrum 500 Imaging System (Upland, CA). The image densitometry analysis was quantified by UN-SCAN-IT gel software (Orem, UT).

### Total RNA extraction and real time-quantitative PCR (RT-qPCR) analysis

Total RNA was isolated from HCFs treated with TNF-α for the indicated time in 10-cm culture dishes with Trizol according to the protocol of the manufacturer. The cDNA obtained from total RNA previously described [33]. RT (reverse transcription) of the first-strand cDNA synthesis was performed with 2 μg of total RNA using oligo-dT as primers in a final volume of 20 μl (2 U/μl RNaseOUT, Cat. 10777–019, and 10 U/μl Moloney murine leukemia virus reverse transcriptase (Cat. 28025–013) from Invitrogen (Carlsbad, CA). The reaction was carried out at 37 °C for 60 min.

Real-time PCR using KAPA PROBE FAST ABI Prism® qPCR kit (KK4705, Kapa Biosystems, Wilmington, MA) was performed with the 7500 Real-Time PCR System (Applied Biosystems, Foster City, CA) using primers and probe mixes for VCAM-1 (Forward: WSO_645213_001; Reverse: WSO_645213_002; Probe: WSO_645213_003; Invitrogen) and endogenous GAPDH (Forward: WSO_724786_001; Reverse: WSO_724786_002; Probe: WSO_724786_003) served as an internal control gene (Invitrogen, Carlsbad, CA). Relative gene expression was determined by the ΔΔCt method, where Ct meant threshold cycle [39]. All experiments were performed in triplicate.

### Plasmid construction, transfection, and luciferase reporter gene assays

For construction of the VCAM-1-Luc plasmid, human VCAM-1 promoter, a region spanning −1716 to −119 bp (kindly provided by Dr. W.C. Aird, Department of Molecular Medicine, Beth Israel Deaconess Medical Center, Boston, MA, USA) was inserted between MluI and XhoI sites of pGL3-basic vector (Promega, Madison, WI). All plasmids were prepared by using QIAGEN plasmid DNA preparation kit. Plasmid (or siRNA) transient transfection of HCFs was performed according to the protocol of Lipofectamine and PLUS reagent (Invitrogen, Carlsbad, CA) with minor modifications. Briefly, HCFs were plated at 3 × 10^5^ cells/ml (1 ml per well) in 12-well culture plates for 24 h, reaching about 80 ~ 90 % confluence. Cells were washed once with PBS and 0.4 ml of serum-free DMEM/F-12 medium was added to each well. The 0.8 μg plasmid DNA (or 20 pmol siRNA) and 1 μl PLUS reagent complex were prepared with serum- and antibiotics-free DMEM/F-12 for 15 min at room temperature. Two μl Lipofectamine™ was mixed gently with serum- and antibiotics-free DMEM/F-12. The mixtures were incubated for 30 min at room temperature. The mixtures (100 μl) were added into the wells. After transfection for 5 h, the cells were washed once with PBS and 0.5 ml of containing 10 % FBS was added and incubated for an additional 19 h. VCAM-1-Luc and κB-Luc (Clontech, Palo Alto, CA) activities in HCFs were determined as previously described [33] using a luciferase assay system (Promega, Madison, WI) according to the manufacturer’s instructions. Firefly luciferase activities were standardized for β-galactosidase activity.

### Cell adhesion assay

HCFs were placed on 6-well culture plates with cover slips and pretreated with the pharmacological inhibitors for 1 h before treatment with TNF-α for 16 h at 37 °C in a humidified 5 % CO_2_ atmosphere. Before the adhesion assay, the HCFs were washed with PBS and then incubated in serum-free DMEM/F-12. THP-1 cells (human acute monocytic leukemia cell line, obtained from ATCC) were maintained in suspension in RPMI-1640 medium supplemented with 10 % FBS, 100 U penicillin, and 100 U streptomycin. Before labeling, THP-1 cells were washed and resuspended in serum-free medium. BCECF/AM (10 μM), a membrane-permeable fluorescent indicator with an Ex/Em = 503/520 nm, was used to label the THP-1 cells in serum free medium for 1 h at 37 °C. After labeling, cells were washed and resuspended in serum free medium at least three times and kept in the dark at room temperature and then the labeled THP-1 cell (2 × 10^6^ cells/ml) were added to HCFs challenged with TNF-α, and cultures were incubated in a CO_2_ incubator for 1 h. Non-adherent THP-1 cells were removed and plates were gently washed twice with PBS. The numbers of fluorescently labeled adherent THP-1 cells were determined by counting four fields per 200× high-power field well using a fluorescence microscope (Zeiss, Axiovert 200 M).

### Statistical analysis

All data were estimated using GraphPad Prism Program (GraphPad, San Diego, CA). Data were expressed as the mean ± SEM and analyzed with a one-way ANOVA followed with Wilcoxon/Kruskal-Wallis test at *p* < 0.05 level of significance.

## Results

### TNF-α induces VCAM-1 expression and monocyte adhesion

**Fig. 1:**
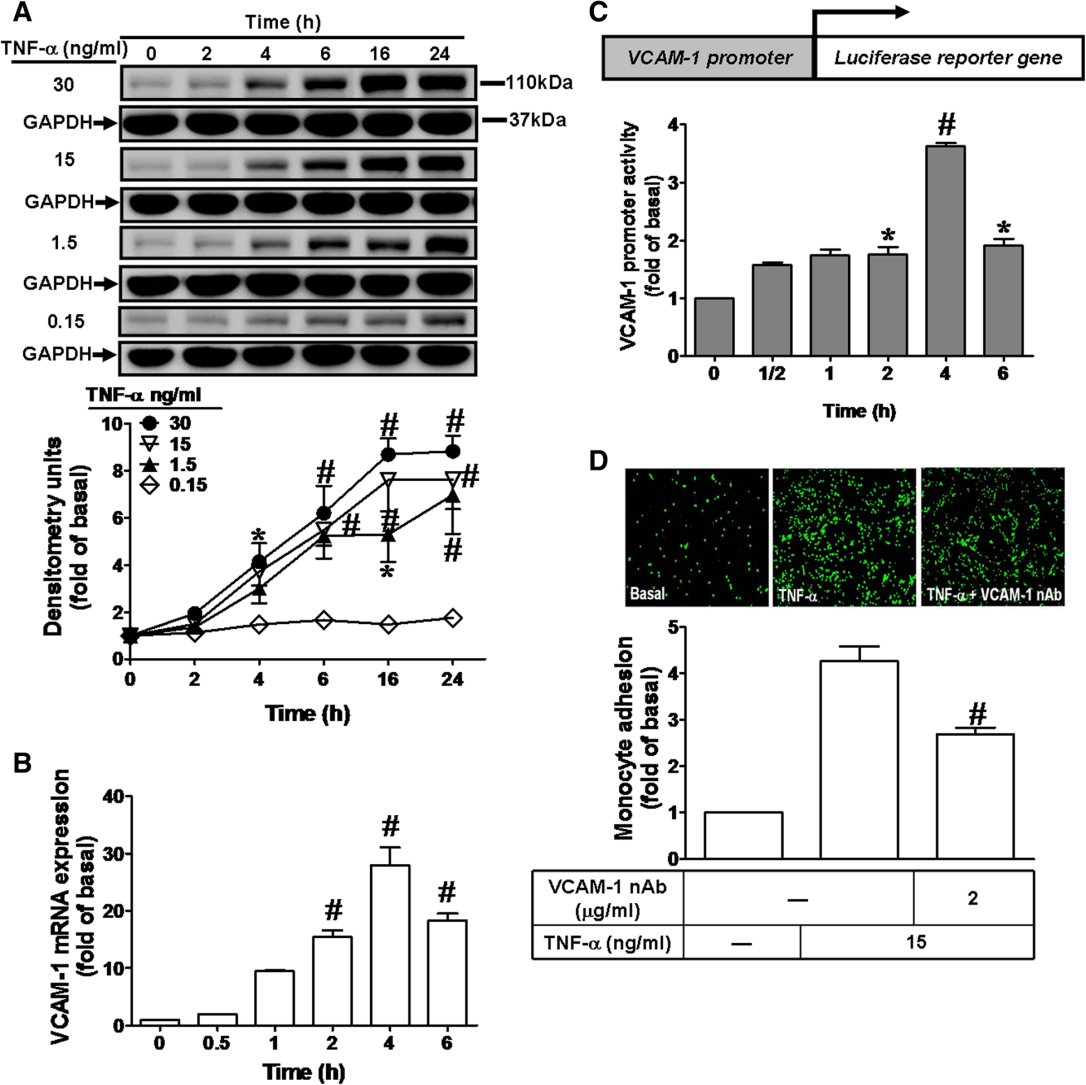
TNF-α induces VCAM-1 expression in HCFs. **a** Time and concentration dependence of TNF-α-induced VCAM-1 expression, HCFs were treated with various concentrations of TNF-α (0.15, 1.5, 15, or 30 ng/ml) for the indicated time intervals. The cell lysates were prepared and blotted using VCAM-1 or GAPDH (as a control) antibody. **b** For VCAM-1 mRNA expression, cells were treated with TNF-α (15 ng/ml) for the indicated times. The VCAM-1 mRNA expression was analyzed by real time-qPCR. **c** For VCAM-1 promoter activity, cells were transiently transfected with a VCAM-1-Luc reporter gene and then treated with TNF-α (15 ng/ml) for the indicated time intervals. The promoter luciferase activity was analyzed and normalized to β-galactosidase activity as described in “Methods”. **d** For adhesion activity, cells were treated with TNF-α for 16 h and washed with PBS prior to the addition of THP-1 monocytes. Moreover, functional expression of VCAM-1 was determined by THP-1 monocyte adhesion assay in the absence or presence of VCAM-1 neutralizing antibody (VCAM-1 nAb, 2 μg/ml). Data are expressed as mean ± SEM of three individual experiments (n = 3). ^*^ *P* < 0.05, ^#^ *P* < 0.01 vs. vehicle (A-C) or TNF-α (**d**) alone

To evaluate the effect of TNF-α on VCAM-1 protein and mRNA expression, HCFs were incubated with various concentrations of TNF-α for the indicated time intervals. As shown in Fig. 1a, TNF-α induced VCAM-1 protein expression in a time- and concentration-dependent manner, with a significant increase within 4 h, a maximal response within 16 h, and sustained over 24 h. There was no change in the levels of house-keeping protein GAPDH served as an internal control. We also found that 30 ng/ml TNF-α induced a maximal VCAM-1 induction within 16–24 h. The concentration of TNF-α (15 ng/ml) was used to induce sub-maximal VCAM-1 expression for the following experiments. To further examine the effect of TNF-α on VCAM-1 mRNA expression, the levels of VCAM-1 mRNA were determined by real time-qPCR. As shown in Fig. 1b, TNF-α (15 ng/ml) time-dependently induced VCAM-1 mRNA accumulation in HCFs. A maximal response was obtained within 4 h and sustained over 6 h during the period of observation. Moreover, TNF-α (15 ng/ml) also stimulated a significant increase in VCAM-1-promoter activity within 2 h and reached a maximum within 4 h (Fig. 1c), suggesting that TNF-α up-regulated VCAM-1 gene expression in HCFs. We further tested the functional activity of expressed VCAM-1 by TNF-α in HCFs, a THP-1 monocyte adhesion assay was performed. As shown in Fig. 1d, TNF-α induced a significant increase of THP-1 monocyte adhesion to TNF-α-challenged HCFs (approximate 4 folds) which was attenuated by pretreatment with VCAM-1 neutralizing antibody (VCAM-1 nAb, 2 μg/ml). These results suggested that induction of VCAM-1 enhances THP-1 monocyte adhesion to the TNF-α-challenged HCFs.

### TNF-α induces *de novo* VCAM-1 protein synthesis via TNF receptor-mediated manner

**Fig. 2:**
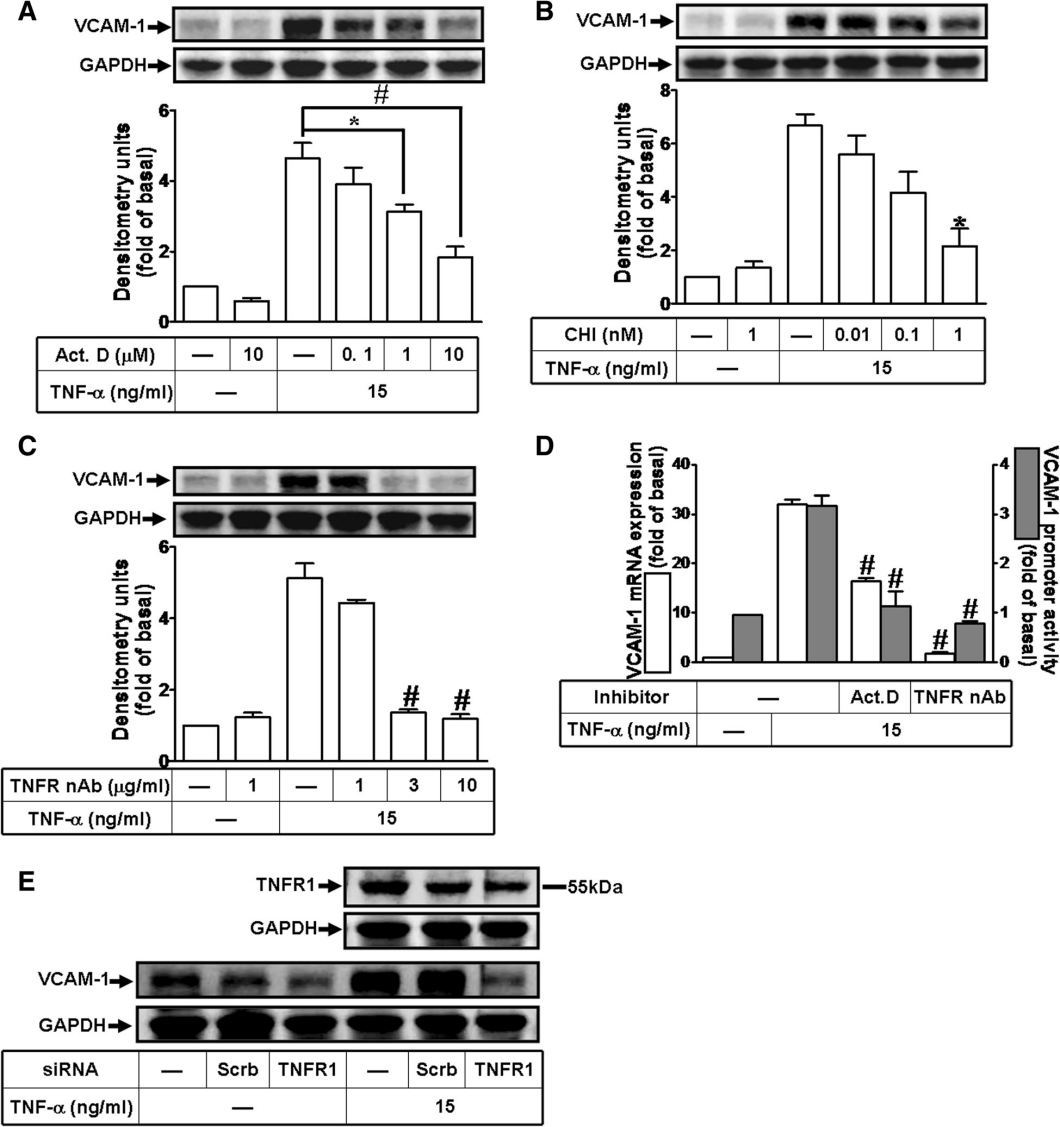
TNF-α induces *de novo* VCAM-1 protein synthesis via TNFR1-mediated manner. **a**, **b** Cells were pretreated with various concentrations of actinomycin D (Act. D, transcription inhibitor) or cycloheximide (CHI, translation inhibitor) and then incubated with 15 ng/ml TNF-α for 16 h. The cell lysates were analyzed by Western blot using a VCAM-1 or GAPDH (as a control) antibody. **c** Involvement of TNFR in TNF-α-induced VCAM-1 expression, cells were pretreated with various concentrations of TNFR neutralizing antibody (TNFR nAb; 1, 3, or 10 μg/ml) for 1 h and then incubated with TNF-α for 16 h. **d** Cells were pretreated with Act. D (10 μM) or TNFR nAb (3 μg/ml) for 1 h and then incubated with TNF-α for 4 h. The VCAM-1 mRNA (open bar) and promoter activity (gray bar) were analyzed by real time-qPCR and promoter assay, respectively. **e** Cells were transfected with siRNA of TNFR1 or scramble (scrb, as a control) for 24 h and then incubated with TNF-α for 16 h. The VCAM-1 protein (**a**, **b**, **c**, **e**) was analyzed by Western blot using respective antibodies. Data are expressed as mean ± SEM of three individual experiments (n = 3). ^*^ *P* < 0.05, ^#^ *P* < 0.01 vs. TNF-α alone

To determine whether the effect of TNF-α on VCAM-1 expression is dependent on de novo protein synthesis, a transcription inhibitor Act. D and a translation inhibitor CHI were used. As shown in Figs. 2a and b, pretreatment with either Act. D or CHI concentration-dependently attenuated TNF-α-induced VCAM-1 expression in HCFs, suggesting that VCAM-1 expression induced by TNF-α is mediated through the transcription and translation. To determine whether TNF-α-induced VCAM-1 expression is mediated through TNF receptor (TNFR), as shown in Fig. 2c, pretreatment with TNFR neutralizing antibody (TNFR nAb) attenuated TNF-α-induced VCAM-1 expression. Moreover, as shown in Fig. 2d, pretreatment with Act. D (10 μM) or TNFR nAb (3 μg/ml) significantly attenuated TNF-α-induced VCAM-1 mRNA expression (open bar) and VCAM-1 promoter activity (gray bar). To confirm the role of TNFR1 in TNF-α-induced VCAM-1 expression, the data showed that transfection with TNFR1 siRNA knocked down TNFR1 protein and attenuated the TNF-α-induced VCAM-1 expression (Fig. 2e), suggesting that TNF-α-induced VCAM-1 expression is mediated through TNFR1-dependent manner in HCFs.

### Involvement of c-Src in TNF-α-induced VCAM-1 expression

**Fig. 3:**
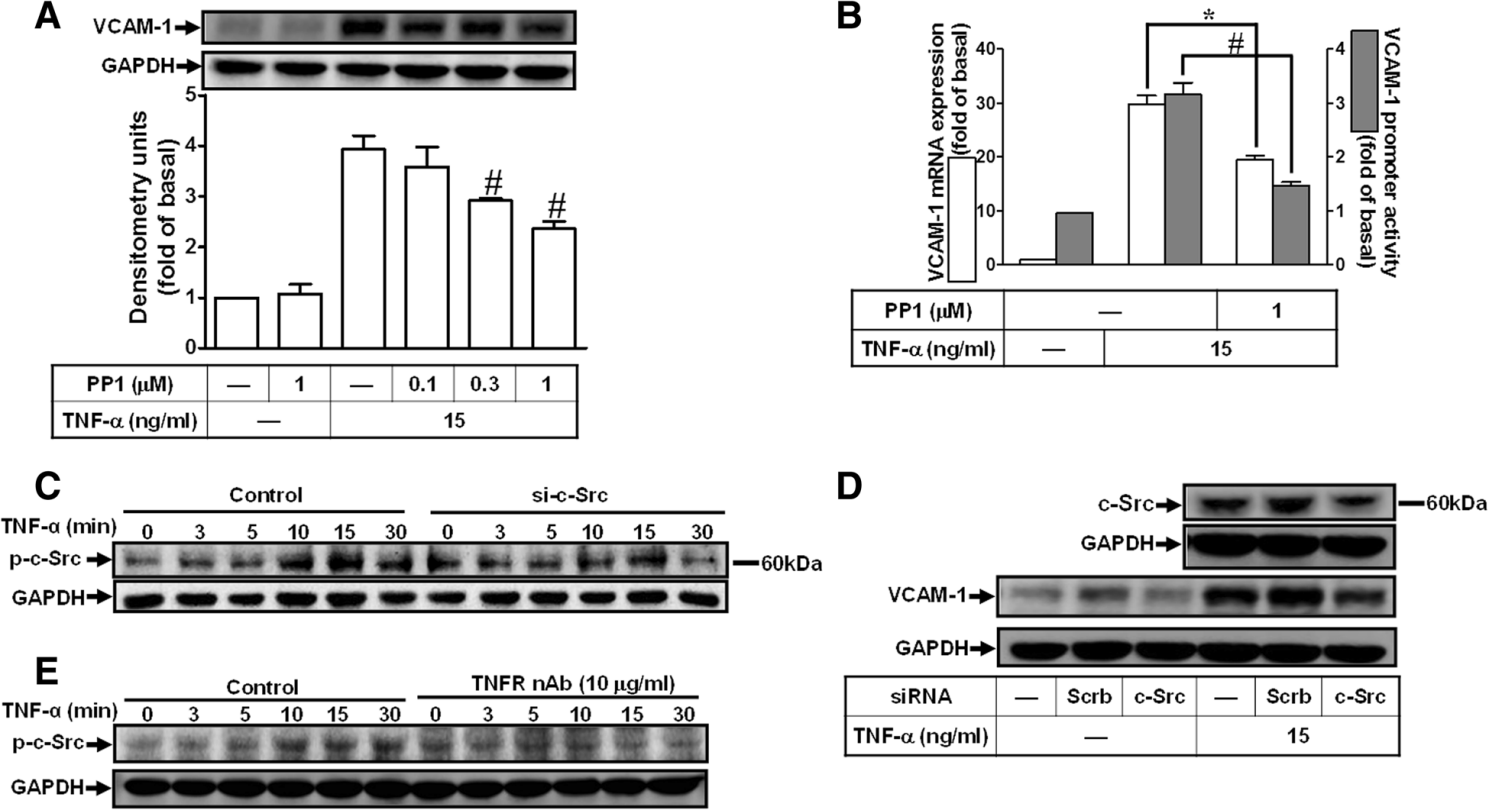
Involvement of c-Src in TNF-α-induced VCAM-1 expression. **a**, **b** Cells were pretreated with (**a**) various concentrations of PP1 (c-Src inhibitor; 0.1, 0.3, or 1 μM) (B) 1 μM PP1 for 1 h and then incubated with TNF-α for 16 h (**a**) or 4 h (**b**, **c**) Time dependence of TNF-α-induced c-Src phosphorylation, cells were treated with TNF-α (15 ng/ml) for the indicated time intervals in the presence or absence of c-Src siRNA. **d** Cells were transfected with siRNA of c-Src or scramble (scrb, as a control) for 24 h and then incubated with TNF-α for 16 h. **e** Cells were treated with TNF-α for the indicated time intervals in the presence or absence of TNFR nAb (10 μg/ml). The VCAM-1 protein (**a**, **d**), and mRNA and promoter activity (**b**) were analyzed by Western blotting, real time-qPCR, and promoter assay, respectively. The c-Src phosphorylation (**c**, **e**) was analyzed by Western blot. Data are expressed as mean ± SEM of three individual experiments (n = 3). ^*^ *P* < 0.05, ^#^ *P* < 0.01 vs. TNF-α alone

TNF-α has been shown to induce expression of adhesion molecules such as VCAM-1 in various cell types [33]. In addition, c-Src is a critical tyrosine kinase to regulate VCAM-1 expression in myoglobin-induced tubular injury [31]. Thus, we investigated whether c-Src involved in TNF-α-induced VCAM-1 expression in HCFs. As shown in Figs. 3a and b, pretreatment with the inhibitor of c-Src (PP1) attenuated TNF-α-induced VCAM-1 protein, mRNA, and promoter activity. To further observe whether TNF-α could stimulate c-Src activation linked to VCAM-1 expression, as shown in Fig. 3c, TNF-α stimulated c-Src phosphorylation in a time-dependent manner in these cells. We further confirmed the effect of c-Src on TNF-α-induced responses, a c-Src siRNA was used. The data showed that transfection with c-Src siRNA markedly knocked down c-Src protein level and attenuated TNF-α-induced c-Src phosphorylation (Fig. 3c) and VCAM-1 expression (Fig. 3d). Moreover, we determined the role of TNFR in these responses, as shown in Fig. 3e, pretreatment with TNFR nAb attenuated TNF-α-stimulated c-Src phosphorylation during the period of observation, suggesting that TNF-α stimulated c-Src phosphorylation via a TNFR-dependent manner. These results suggested that TNF-α-induced VCAM-1 expression is mediated through a c-Src-mediated pathway in HCFs.

### c-Src-dependent transactivation of EGFR is involved in TNF-α-induced VCAM-1 expression

**Fig. 4:**
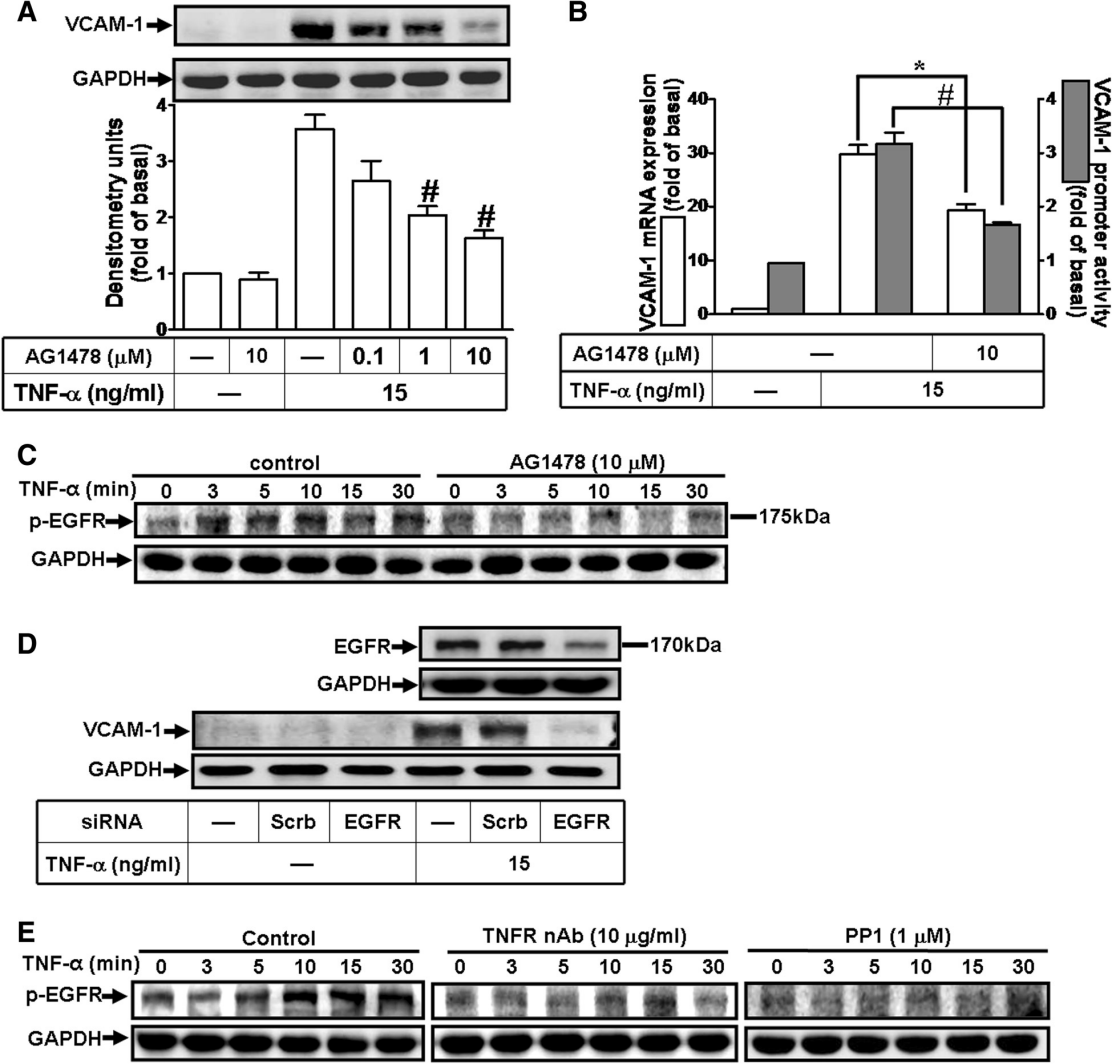
TNF-α-induced VCAM-1 expression is mediated through c-Src-dependent transactivation of EGFR. **a**, **b** Cells were pretreated with various concentrations of AG1478 (EGFR inhibitor; 0.1, 1, or 10 μM) for 1 h and then incubated with TNF-α for 16 h (**a**) or 4 h (**b**). **c** Time dependence of TNF-α-induced EGFR phosphorylation, cells were treated with TNF-α (15 ng/ml) for the indicated time intervals in the presence or absence of AG1478 (10 μM). **d** Cells were transfected with siRNA of EGFR or scramble (scrb, as a control) for 24 h and then incubated with TNF-α for 16 h. **e** Cells were treated with TNF-α for the indicated time intervals in the presence or absence of TNFR nAb (3 μg/ml) or PP1 (1 μM). The VCAM-1 protein (**a**, **d**), and mRNA and promoter activity (**b**) were analyzed by Western blotting, real time-PCR, and promoter assay, respectively. The EGFR phosphorylation (**c**, **e**) was analyzed by Western blot. Data are expressed as mean ± SEM of three individual experiments (n = 3). ^*^ *P* < 0.05, ^#^ *P* < 0.01 vs. TNF-α alone

Several growth factor receptors such as EGFR activation have been shown to be regulated by c-Src leading to induction of various genes [40]. Thus, we investigated the role of EGFR in TNF-α-induced VCAM-1 expression in HCFs. As shown in Figs. 4a and b, pretreatment with an EGFR inhibitor AG1478 concentration-dependently inhibited TNF-α-induced VCAM-1 protein, mRNA, and promoter activity. To determine whether TNF-α could stimulate phosphorylation of EGFR linked to VCAM-1 expression, as shown in Fig. 4c, TNF-α time-dependently stimulated EGFR phosphorylation which was attenuated by pretreatment with AG1478 (10 μM) during the period of observation. We further confirmed the role of EGFR in TNF-α-induced VCAM-1 expression, the results showed that transfection with EGFR siRNA knocked down EGFR protein level and attenuated TNF-α-induced VCAM-1 expression (Fig. 4d). We also investigated the role of TNFR-mediated c-Src cascade in TNF-α-stimulated EGFR phosphorylation. As shown in Fig. 4e, pretreatment with TNFR nAb or PP1 attenuated TNF-α-stimulated EGFR phosphorylation during the period of observation. These results suggested that c-Src-dependent EGFR transactivation contributes to TNF-α-induced VCAM-1 expression in HCFs.

### TNF-α induces VCAM-1 expression via PI3K/Akt cascade

**Fig. 5:**
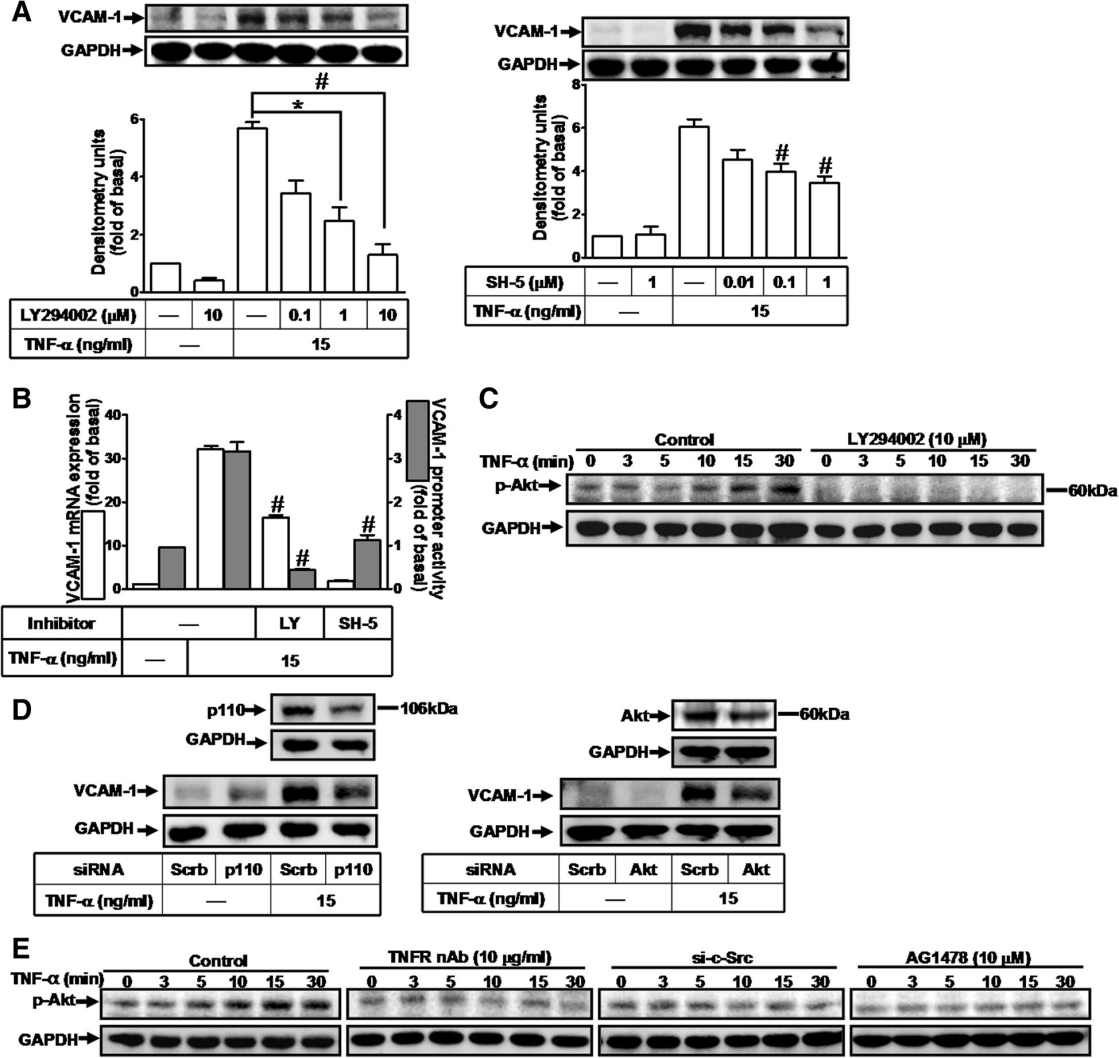
TNF-α induces VCAM-1 expression via PI3K/Akt cascade. **a**, **b** Cells were pretreated with various concentrations of LY294002 (PI3K inhibitor; 0.1, 1, or 10 μM) or SH-5 (Akt inhibitor; 0.01, 0.1, or 1 μM) for 1 h and then incubated with TNF-α for 16 h (**a**) or 4 h (b). **c** Time dependence of TNF-α-induced Akt phosphorylation, cells were treated with TNF-α (15 ng/ml) for the indicated time intervals in the presence or absence of LY294002 (10 μM). **d** Cells were transfected with siRNA of p110 (**a** PI3K subunit), Akt, or scramble (scrb, as a control) for 24 h and then incubated with TNF-α for 16 h. **e** Cells were treated with TNF-α for the indicated time intervals in the presence or absence of TNFR nAb (10 μg/ml), c-Src siRNA, or AG1478 (10 μM). The VCAM-1 protein (**a**, **d**), and mRNA and promoter activity (**b**) were analyzed by Western blotting, real time-PCR, and promoter assay, respectively. The Akt phosphorylation (**c**, **e**) was analyzed by Western blot. Data are expressed as mean ± SEM of three individual experiments (n = 3). ^*^ *P* < 0.05, ^#^ *P* < 0.01 vs. TNF-α alone

The PI3K/Akt signaling cascade, the direct downstream molecules of EGFR, plays a key role in various physiological and pathological processes. Previous studies have indicated that PI3K/Akt pathway is involved in VCAM-1 induction by TNF-α in various cell types [40, 41]. In this study, we investigated the effects of PI3K (LY294002) and Akt (SH-5) inhibitors on TNF-α-induced VCAM-1 expression. As shown in Figs. 5a and b, pretreatment with LY294002 or SH-5 markedly attenuated TNF-α-induced VCAM-1 protein, mRNA, and promoter activity. To determine whether TNF-α stimulates activation of Akt, the data showed that TNF-α time-dependently stimulated Akt phosphorylation which was inhibited by pretreatment with LY294002 (10 μM) during the period of observation (Fig. 5c). To confirm the roles of PI3K/Akt in TNF-α-induced VCAM-1 expression in HCFs, as shown in Fig. 5d, transfection with siRNA for p110 (a PI3K subunit) or Akt knocked down p110 or Akt protein, respectively, and significantly attenuated TNF-α-induced VCAM-1 expression. We further determined the role of c-Src-dependent EGFR signaling pathway in TNF-α-stimulated Akt phosphorylation. As shown in Fig. 5e, pretreatment with TNFR nAb, c-Src siRNA, or AG1478 markedly attenuated TNF-α-induced Akt phosphorylation in HCFs. These results indicated that TNF-α-induced VCAM-1 expression is mediated through a c-Src-dependent transactivation of EGFR-PI3K/Akt-dependent signaling pathway in HCFs.

### NF-κB is required for TNF-α-induced VCAM-1 expression

**Fig. 6:**
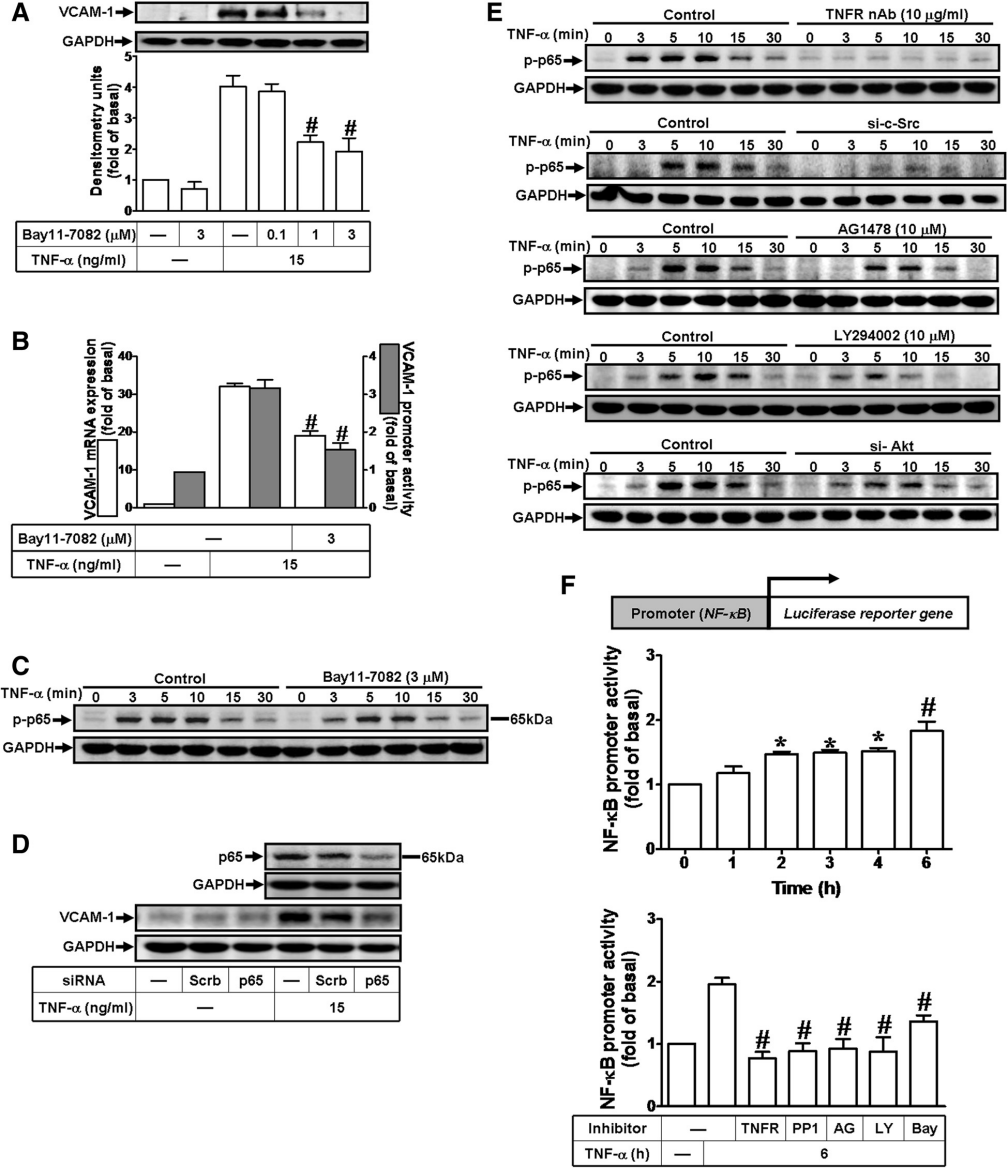
The NF-κB is required for TNF-α-induced VCAM-1 expression. **a**, **b** Cells were pretreated with various concentrations of Bay11-7082 (NF-κB inhibitor; 0.1, 1, or 3 μM) for 1 h and then incubated with TNF-α for 16 h (**a**) or 4 h (**b**). **c** Time dependence of TNF-α-induced p65 NF-κB phosphorylation, cells were treated with TNF-α (15 ng/ml) for the indicated time intervals in the presence or absence of Bay11-7082 (3 μM). **d** Cells were transfected with siRNA of p65 or scramble (scrb, as a control) for 24 h and then incubated with TNF-α for 16 h. **e** Cells were treated with TNF-α for the indicated time intervals in the presence or absence of TNFR nAb (10 μg/ml), c-Src siRNA, or AG1478 (10 μM), LY294002 (10 μM), or Akt siRNA. **f** Time dependence of TNF-α-stimulated NF-κB transcription activity, cells were transfected with a NFκB-luciferase reporter gene and then exposed to TNFα for the indicated time intervals (upper panel). Moreover, the transfected cells were pretreated with the inhibitor of NF-κB (Bay11-7082), TNFR nAb, c-Src (PP1), EGFR (AG1478), or PI3K (LY294002) for 1 h and then incubated with TNF-α for 6 h (lower panel). The VCAM-1 protein (**a**, **d**), mRNA (**b**), and promoter activity (**b**, **f**) were analyzed by Western blotting, real time-qPCR, and promoter assay, respectively. The Akt phosphorylation (**c**, **e**) was analyzed by Western blot. Data are expressed as mean ± SEM of three individual experiments (n = 3). ^*^ *P* < 0.05, ^#^ *P* < 0.01 vs. TNF-α alone

Inflammatory responses following stimulation with cytokines such as TNF-α are highly dependent on activation of the transcription factor NF-κB. Moreover, TNF-α has been shown to induce expression of various genes such as VCAM-1 through NF-κB in several cell types [33]. Thus, the involvement of NF-κB in VCAM-1 induction by TNF-α in HCFs was confirmed by using an NF-κB pharmacological inhibitor Bay11-7082. As shown in Fig. 6a, pretreatment with Bay11-7082 caused a concentration-dependent attenuation of VCAM-1 expression induced by TNF-α in HCFs. We further investigated the effects of Bay11-7082 on TNF-α-induced VCAM-1 mRNA and promoter activity. As shown in Fig. 6b, pretreatment with Bay11-7082 (3 μM) attenuated TNF-α-induced VCAM-1 mRNA and promoter activity. To determine whether TNF-α stimulates activation of NF-κB, the data showed that TNF-α time-dependently stimulated p65 (**a** subunit of NF-κB) phosphorylation which was attenuated by pretreatment with Bay11-7082 during the period of observation (Fig. 6c). Furthermore, to confirm the role of NF-κB in TNF-α-induced VCAM-1 expression, as shown in Fig. 6d, transfection with p65 siRNA knocked down p65 protein level and attenuated TNF-α-induced VCAM-1 expression, suggesting that NF-κB is essential for these responses. We further determined whether involvement of c-Src-dependent EGFR/PI3K/Akt signaling pathway in TNF-α-stimulated p65 phosphorylation in HCFs. As shown in Fig. 6e, pretreatment with TNFR nAb, c-Src siRNA, AG1478, LY294002, or Akt siRNA markedly attenuated TNF-α-stimulated p65 phosphorylation, suggesting that TNFR/c-Src/EGFR/PI3K/Akt cascade is involved in TNF-α-stimulated p65 phosphorylation in HCFs. Moreover, to determine whether TNF-α enhances the transcriptional activity of NF-κB, as shown in Fig. 6f, we observed that TNF-α enhanced NF-κB transcriptional activity in a time-dependent manner and a maximal response was achieved within 6 h (upper panel), which was markedly inhibited by pretreatment with TNFR nAb, PP1, AG1478, LY294002, or Bay11-7082 (lower panel). Taken together, these data demonstrated that TNF-α induces NF-κB-dependent VCAM-1 expression via a TNFR/c-Src/EGFR/PI3K/Akt signaling pathway in HCFs.

### TNF-α promotes monocyte adhesion to HCFs through a c-Src-dependent EGFR/PI3K/Akt/NF-κB pathway

**Fig. 7:**
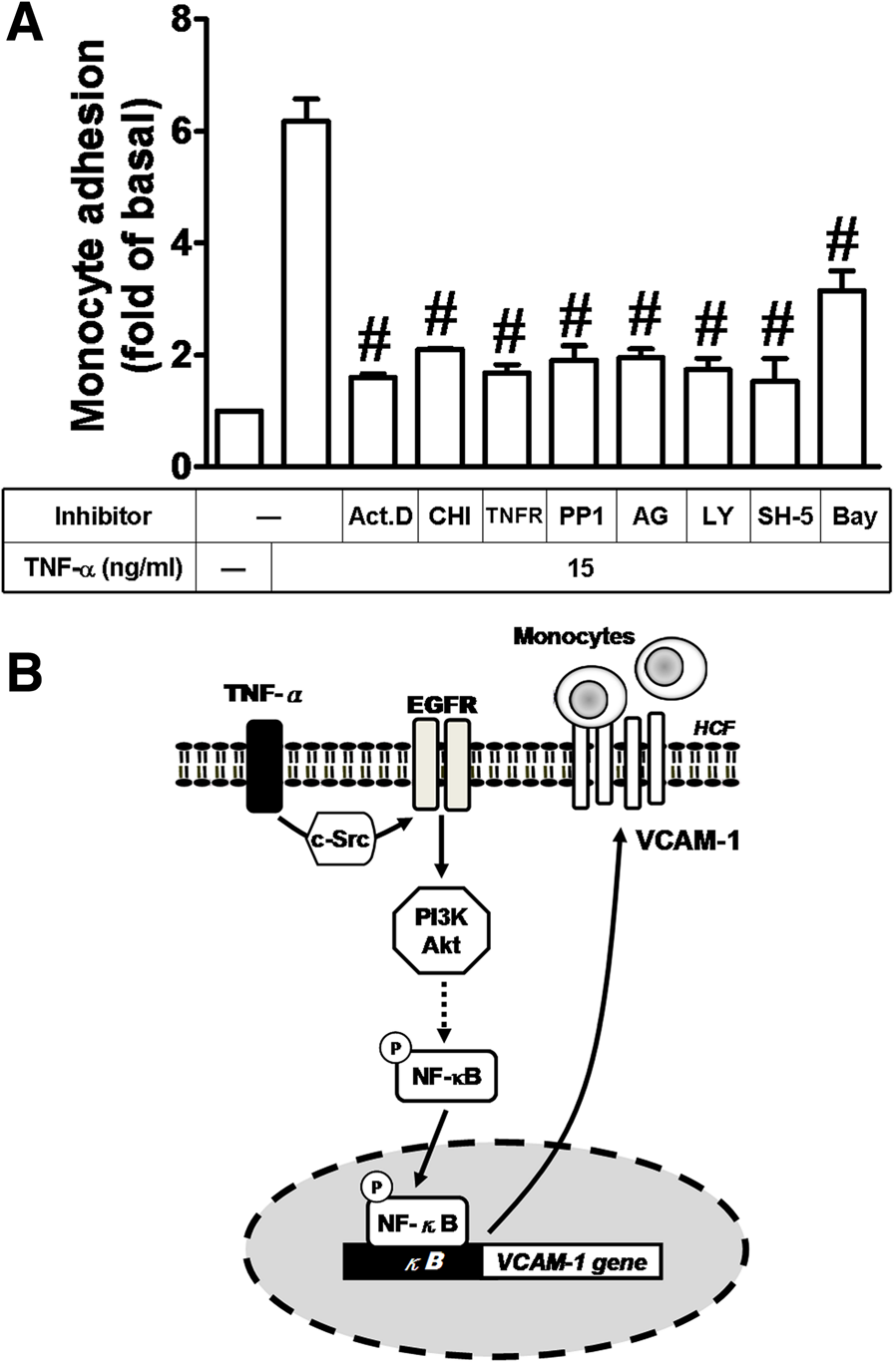
Up-regulated VCAM-1 by TNF-α contributes to enhancement of monocyte adhering to HCFs. **a** For adhesion activity, cells were pretreated with Act. D (transcription inhibitor, 10 μM), CHI (translation inhibitor, 1 μM), TNFR-nAb (10 μg/ml), PP1 (c-Src, inhibitor, 1 μM), AG1478 (EGFR inhibitor, 10 μM), LY294002 (PI3K inhibitor, 10 μM), SH-5 (Akt inhibitor, 1 μM), or Bay11-7082 (NF-κB inhibitor, 3 μM) for 1 h and then incubated with TNF-α (15 ng/ml) for 16 h prior to the addition of THP-1 monocytes. The adhesion activity was determined by cell adhesion assay as described in Fig. 1. Data are expressed as mean ± SEM of at least three individual experiments (n = 3). ^#^ *P* < 0.01 vs. TNF-α alone. **b** Schematic presentation of the summary of this work. In HCFs, TNF-α/TNFR1-mediated signaling pathways linked to up-regulation of VCAM-1. TNF-α induces VCAM-1 expression via c-Src-dependent transactivation of EGFR/PI3K/Akt cascade linking to p65 NF-κB transcription activity. Finally, TNF-α-up-regulated VCAM-1 contributes to enhancement of monocyte adhering to HCFs

Our previous data (Fig. 1d) have shown that TNF-α induces VCAM-1 expression and then enhancing monocyte adhesion to HCFs. Subsequently, we further investigated whether these signaling molecules investigated above are involved in enhancing monocyte adhesion to HCFs challenged with TNF-α and their respective pharmacological inhibitors were used. As shown in Fig. 7a, pretreatment with Act. D (10 μM), CHI (1 μM), TNFR nAb (3 μg/ml), PP1 (1 μM), AG1478 (10 μM), LY294002 (10 μM), SH-5 (1 μM), or Bay11-7082 (3 μM) markedly blocked the adhering of THP-1 to TNF-α-challenged HCFs. These results indicated that TNF-α-induced VCAM-1 expression is mediated through a c-Src-dependent EGFR/PI3K/Akt/NF-κB pathway which also contributes to enhancement of monocyte adhering to these TNF-α-induced HCFs.

## Discussion

Up-regulation of adhesion molecules on the surface of the heart cells may play a key role in recruitment and infiltration of leukocytes at sites of inflammation in heart inflammatory disorders [8, 23, 24, 26]. Cardiac fibroblasts play critical role in the development of cardiac fibrosis [4, 10, 11, 42]. Previous reports indicate that TNF-α-treatment can induce VCAM-1 expression in rat primary cardiac fibroblasts [43] and mediate the monocyte adhesion [44, 45]. We found that VCAM-1 nAb can reduce the monocyte adhesion (about 37 %) on HCFs challenged with TNF-α (Fig. 1d). However, TNFR nAb treatment can reduce the monocyte adhesion on HCFs about 73 % (Fig. 7a). We suggest that TNF-α induces not only VCAM-1 but also other adhesion molecules on HCFs which contribute to monocyte adhesion.

Previous study indicates that serum TNF-α concentrations of chronic heart failure patients (4.5 ± 0.5 pg/ml) are higher than normal (2.6 ± 0.4 pg/ml) [46]. However, treatments with these concentrations could not induce a significant gene expression in our system (data not shown). To obtain the optimal VCAM-1 expression, the higher concentrations of TNF-α were used to challenge HCFs. TNF-α has been confirmed to induce the expression of VCAM-1 in synovial fibroblasts [33]. Moreover, TNF-α has also been shown to activate various signals including MAPKs in several cell types [6, 33], but the intracellular signaling mechanisms leading to VCAM-1 expression remain unclear in HCFs. Transactivation of the receptor tyrosine kinases (RTKs), EGFR especially, has been shown to occur in response to several cytokines such as TNF-α [47], which might contribute to various cellular functions [35]. Although transactivation of EGFR by GPCR agonists has been well studied [35, 48], the signaling mechanisms by which TNF-α-stimulated transactivation of EGFR in HCFs remain unclear.

In this study, up-regulation of VCAM-1 by TNF-α is mediated through transactivation of c-Src/EGFR which were attenuated by their respective inhibitors (PP1 and AG1478) and siRNAs transfection, suggesting the involvement of c-Src/EGFR in these responses (Figs. 3 and 4). In addition, we found that EGFR was a downstream component of c-Src, since TNF-α-induced EGFR phosphorylation was attenuated by PP1 (Fig. 4e), whereas AG1478 had no effect on c-Src phosphorylation (data not shown), and subsequent up-regulation of VCAM-1 and adhesion of monocytes (Fig. 7a). These results suggested that transactivation of EGFR plays a critical role in TNF-α-induced VCAM-1 expression and monocyte adhesion, consistent with previous report showing the involvement of EGFR transactivation in TNF-α-induced VCAM-1 expression in human tracheal smooth muscle cells (HTSMCs) [40].

The PI3K/Akt cascade is the downstream components of EGFR activation, which is implicated in the pathogenesis of inflammatory responses [49, 50] through the induction of inflammatory gene expression [40, 51]. PI3K could initiate a series of events that lead to Akt activation [52]. Moreover, TNF-α-stimulated activation of PI3K/Akt pathway has been reported in various cell types [6, 22]. As expected, our results also showed that both the inhibitors of PI3K (LY294002) and Akt (SH-5) could significantly attenuate the TNF-α-induced VCAM-1 expression (Fig. 5a) and monocyte adhesion (Fig. 7a). We further confirmed this hypothesis by the results that transfection with p110 siRNA (a PI3K subunit) or Akt siRNA also attenuated the TNF-α-induced VCAM-1expression (Fig. 5d). Altogether, these results suggested that VCAM-1 induction by TNF-α is mediated through a PI3K/Akt-dependent cascade in HCFs, consistent with previous study that PI3K/Akt cascade may be an important pathway in regulating VCAM-1 expression by TNF-α in various cell types [40, 41]. Moreover, TNF-α-stimulated Akt phosphorylation was attenuated by the inhibitors of TNFR nAb, c-Src siRNA, EGFR (AG1478) (Fig. 5e) and PI3K (LY294002) (Fig. 5c). These results indicated that TNF-α-induced VCAM-1 expression may be sequentially mediated through a c-Src-dependent EGFR/PI3K/Akt cascade in HCFs.

Inflammatory responses following exposure to cytokines are highly dependent on activation of NF-κB which plays an important role in expression of several inflammatory genes [17, 18, 53]. NF-κB activity is increased and critically implicates in the development of cardiac fibrosis and pathologic cardiac remodeling. Moreover, NF-κB is a key regulator in myofibroblast differentiation [54]. Our previous study also demonstrated that c-Src-dependent NF-κB activation is essential for the expression of VCAM-1 induced by TNF-α in HTSMCs, synovial fibroblasts and osteoblast-like MC3T3-E1 cells [33, 34, 47]. In the present study, the role of NF-κB in TNF-α-induced VCAM-1 expression is confirmed by an NF-κB inhibitor (Bay11-7082, Fig. 6a) and p65 siRNA transfection (Fig. 6d), indicating that NF-κB involves in TNF-α-induced VCAM-1 expression. Moreover, we found that the increase in p65 NF-κB phosphorylation correlates with an increase of NF-κB transcriptional activity induced by TNF-α (Figs. 6e and f, upper panel). TNF-α-stimulated p65 phosphorylation and transcriptional activity is attenuated by PP1, AG1478, LY294002, and Bay11-7082 or transfection with c-Src and Akt siRNAs (Figs. 6e and f, lower panel), suggesting that TNF-α-stimulated NF-κB activation is mediated through a c-Src-dependent transactivation EGFR/PI3K/Akt cascade in HCFs. These findings are consistent with several reports indicating that activation of NF-κB is mediated via EGFR/PI3K/Akt in various cell types [55, 56]. However, studies on the role of the PI3K/Akt pathway in NF-κB-dependent gene expression are controversial. Our previous study shows that TNF-α-induced MMP-9 expression via NF-κB activation is independent on the PI3K/Akt cascade in HTSMCs [21]. The recent reports also indicate that MMP-9 is a responsive gene of NF-κB and AP-1 [57] and the necrotic cardiomyocytes can release heat-labile proinflammatory signal to activate NF-κB in cardiac fibroblasts [58].

The infiltration of immune cells is due to up-regulation of adhesion molecules including ICAM-1 and VCAM-1 [23, 24, 26] which contribute to several chronic inflammatory diseases including cardiovascular diseases. Moreover, the elevated levels of ICAM-1 and VCAM-1 are often found in the serum of patients with non-ischemic heart failure. These raised serum levels of adhesion molecules correlate with inflammatory infiltrates in the myocardial tissue [27]. The VCAM-1, which exhibits low to negligible expression on un-stimulated endothelial cells, can be profoundly up-regulated by cytokines. The cardiac fibroblasts are versatile cells with the potential to activate an array of genes that are able to initiate and propagate inflammation in heart diseases. Up-regulation of VCAM-1 and TNF-α has been shown in the cardiac vascular endothelium from patients with chronic heart failure [27, 30]. However, the effects of TNF-α-induced VCAM-1 expression between cardiac fibroblasts and immune cells remain unclear. In this study, we demonstrated that TNF-α enhanced monocyte adhesion through up-regulation of VCAM-1, as a marker of immune cell infiltration (Fig. 1d). Moreover, these signaling components of VCAM-1 induction by TNF-α were involved in TNF-α-enhanced monocyte adhesion attenuated by their respective pharmacological inhibitors (Fig. 7a). These results indicated that TNF-α-induced VCAM-1 expression via a c-Src-dependent transactivation of EGFR/PI3K/Akt links to NF-κB pathway which increases the monocytes adhered to TNF-α-challenged HCFs. These results are consistent with reports indicating that TNF-α enhanced immune cells adhesion via increased VCAM-1 expression in RASFs or HTSMCs [33, 40]. Therefore, infiltration of immune cells into myocardium exerts bidirectional cross talk with HCFs which can be mediated by paracrine signals, direct cell-cell interactions, and indirect interaction via ECM. Both cell types and their cross talk are important determinants of structural, mechanical characteristics in the healthy and remodeled myocardium [59]. Cardiac fibroblasts play many of roles in cardiac development and remodeling. HCFs are the predominant type of cells involved in ECM turnover and deposition in the heart [60], which leads to excessive deposition of ECM and fibroblast accumulation and results in distorted organ architecture and function.

## Conclusions

In summary, we reported here that TNF-α induced the expression of VCAM-1 gene in HCFs. The TNFR1, c-Src, EGFR, PI3K/Akt, and NF-κB cooperatively mediated these effects of TNF-α. Based on the observations from literatures and our findings, Fig. 7b depicts a model for the signaling mechanisms implicated in TNF-α-induced VCAM-1 expression in HCFs. These findings suggest that TNF-α-induced VCAM-1 expression might play a critical role in heart inflammatory disorders mediated through c-Src-dependent transactivation of EGFR, PI3K/Akt, and NF-κB signaling pathways in HCFs. The results provide new insights into the mechanisms of TNF-α action on HCFs to up-regulate the VCAM-1 expression and then amplified the inflammatory responses, supporting the hypothesis that TNF-α may play a key role in the development of cardiac diseases.

## Abbreviations

TNF-α: tumor necrosis factor-α
VCAM-1: vascular cell adhesion molecule-1
HCFs: human cardiac fibroblasts
NF-κB: Nuclear factor-κB
EGFR: EGF receptor
DMEM/F-12: Dulbecco’s modified Eagle’s medium/Ham’s nutrient mixture F-12
FBS: fetal bovine serum
Act. D: Actinomycin D
CHI: cycloheximide
ECL: enhanced chemiluminescence
BCA: bicinchoninic acid
PMNs: polymorphonuclear cells
PBS: phosphate-buffered saline
GPCR: G protein-coupled receptor
siRNA: small interfering RNA
PCR: polymerase chain reaction
RTKs: receptor tyrosine kinases
TNFR1: TNF receptor 1
XTT: (sodium 3′-[1-(phenylamino-carbonyl)-3,4-tetrazolium]-bis(4-methoxy-6-nitro) benzene sulphonic acid hydrate
